# A case report of acquired methemoglobinemia rescued by veno-venous extracorporeal membrane oxygenation

**DOI:** 10.1097/MD.0000000000025522

**Published:** 2021-04-16

**Authors:** Yu-Hsuan Lien, You-Cian Lin, Robert Jeenchen Chen

**Affiliations:** a Division of Cardiovascular Surgery, Department of Surgery, China Medical University Hospital, Taichung, Taiwan; b Division of Cardiac Surgery, Department of Surgery, Ohio State University Wexner Medical Center, Columbus, Ohio.

**Keywords:** case report, methemoglobinemia, veno-venous extracorporeal membrane oxygenation

## Abstract

**Rationale:**

Severe methemoglobinemia (Met-Hb) is rare. The delayed diagnosis and treatment often cause further damage. The management of cellular hypoxemia is challenging and the use of extra-corporeal membrane oxygenation (ECMO) has never been reported.

**Patient concerns:**

The young patient, healthy with unremarkable past medical history, was sent to emergency room with out-of-hospital circulatory arrest (OHCA) and severe generalized cyanosis. His family reported he ingested sodium nitrite accidentally.

**Diagnoses:**

After successful resuscitation and return of spontaneous circulation (ROSC), the paradoxically normal arterial blood gas (ABG) with the unusual brownish blood led to the suspicion of Met-Hb. The lab test confirmed it and showed a very high level of 80%.

**Interventions:**

Because of recovered and normal cardiac function, we placed veno-venous extracorporeal membrane oxygenation (VV-ECMO) for tissue hypoxemia in addition to exchange transfusion, vitamin C, and methylene blue.

**Outcomes:**

Met-Hb blood level dropped rapidly. After vigorous rehabilitation for weeks, the patient was able to be discharged home without major neurological sequela.

**Lessons:**

VV-ECMO can hyper-oxygenate the hypoxemic tissue regardless the etiology and minimize hypoxemia-reperfusion injury while awaiting the definite diagnosis and therapy.

## 1 Introduction

Methemoglobinemia (Met-Hb) can be congenital or acquired. Most cases of Met-Hb are acquired, resulting from increased methemoglobin formation induced by various exogenous substances.^[4,5,7]^ The antidote currently recommended for severe Met-Hb is methylene blue.^[4–7]^ Ascorbic acid (Vitamin C), exchange transfusion, and hyperbaric oxygen have been reported to be beneficial according to the guidelines and literature.^[4–7]^ However, there were no literature reports mentioned the role of extracorporeal membrane oxygenation as a bridging rescue in this emergent scenario. Here we present a Met-Hb case treated with venous-venous extracorporeal membrane oxygenation (VV-ECMO) as rescue while awaiting antidote infusion and successfully survived without neurological deficits.

## 2 Case presentation

This 23-year-old man ingested sodium nitrite accidentally at home. He was sent to emergency department presenting as out-of-hospital cardiac arrest (OHCA) and generalized cyanosis. Cardiopulmonary resuscitation (CPR) was initiated by the emergency technical technicians (EMT) on the ambulance. Return of spontaneous circulation (ROSC) was achieved 10 minutes after arriving the emergency room with the efforts of physicians and team members. Because of the brownish-colored blood with the paradoxically normal PaO_2_ levels by the arterial blood gas (ABG), Met-Hb was highly suspected and then confirmed by laboratory data (Met-Hb = 80%). For the status of OHCA with unusual presentation of blood gas physiology, cardiovascular surgeons were consulted for the opinions of ECMO. With stable hemodynamics without the need of strong inotropes and pressors, indicating recovered and normal cardiac function, we decided to implant VV-ECMO via both femoral veins at the bedside, while awaiting the dispensing of methylene blue from the pharmacy.

**Figure 1:**
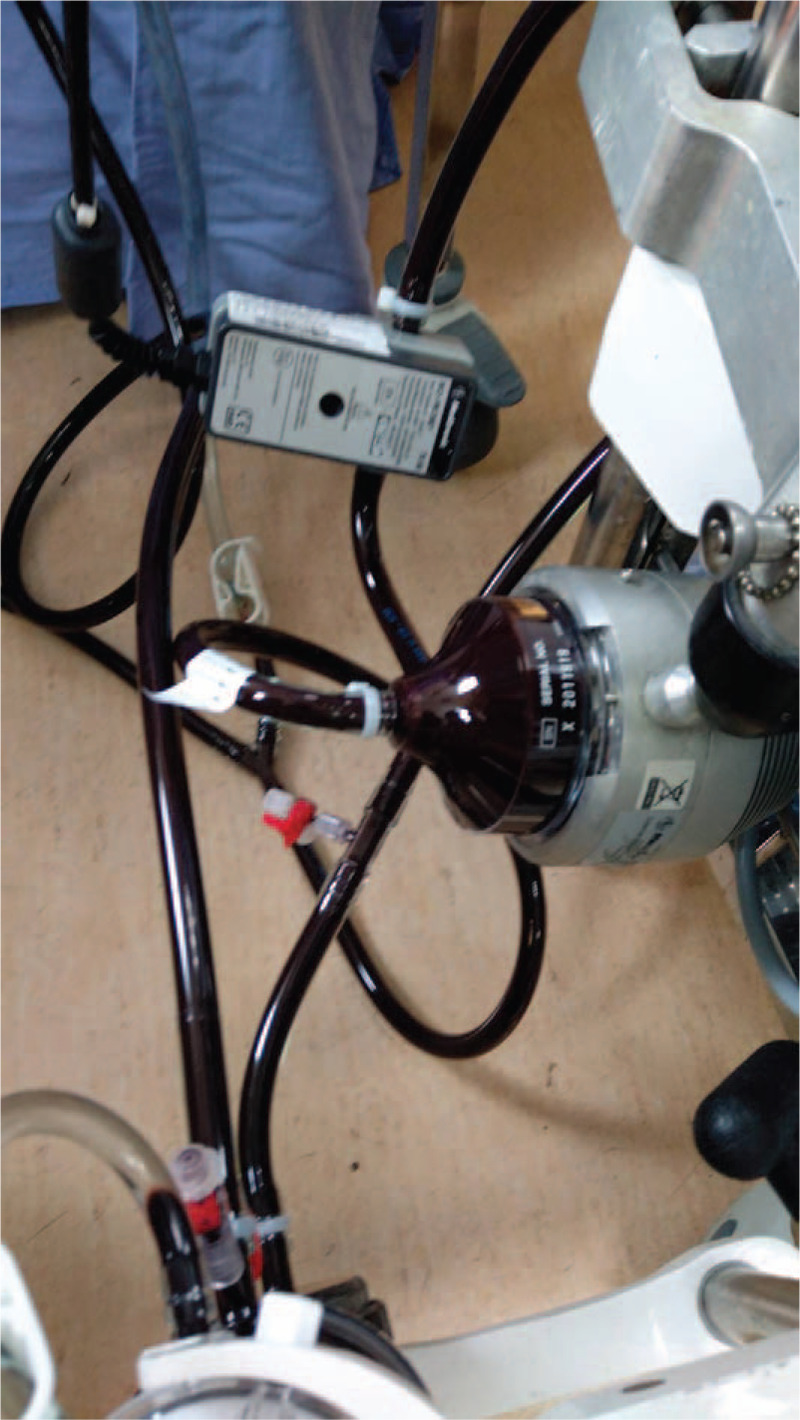
The hyper-oxygenated blood of methemoglobinemia still showed chocolate-brownish color in the ECMO cannula. ECMO = extra-corporeal membrane oxygenation.

The VV-ECMO settings were as following: oxygenator FiO_2_ 100%, sweep 2 L/min titratable with PaCO_2_ from patient's ABG, pump flow 4 L/min, with heparin infusion that kept activated clotting time (ACT) from 180 to 200 seconds. The ventilator setting was adjusted from maximum to FiO_2_ 60% and positive end expiratory pressure (PEEP) of 10 cm H_2_O. The subsequent ABG showed PaO_2_ of 550 mm Hg, although the blood color still remained chocolate-brownish (Fig. 1). Therapies were given as intravenous high-dose vitamin C (1000 mg) and exchange transfusion. Chest computed tomography angiography (CTA) excluded aortic dissection or pulmonary embolism. Four hours after the ingestion of sodium nitrite, methylene blue was infused (100 mg) intravenously. Met-Hb dropped from 80% to 7.5% 1 hour later and then further down to 1.4%.

Clinical cyanosis improved and the patient was sent to intensive care unit for further management. For coma with intact pupil light reflexes, hypothermia therapy began with the target temperature of 33 °C and its course was completed in 2 days. VV-ECMO was weaned after the hypothermia therapy completed 3 days later. The parameters that dictated the ECMO weaning had to show adequate oxygenation without ECMO, such as absence of Met-Hb in blood, clear lung in the chest X-ray, and sufficient tissue perfusion and good ABG with ECMO with zero sweep or low sweep with low ECMO FiO_2_. Brain magnetic resonance imaging showed transient splenial lesion in the genu and splenium of corpus callosum.

The patient woke up 5 days later with post-shock syndrome and generalized weakness that all improved after relevant treatments. The patient had a difficult ventilator weaning but extubation finally succeeded on the 4th week without tracheostomy. After vigorous rehabilitation, the patient was discharged home on the 2nd month, with the cerebral performance category (CPC) score of 1, showing no major neurological sequelae. At the outpatient clinic, the patient has signed the informed consent for publication of this case report.

## 3 Discussion

This unusual case of Met-Hb demonstrated the usual role of ECMO: bridging to decision, therapy, or recovery. When an unknown scenario occurs, the ECMO support stops the crisis escalation and the next step can have time to be optimized, just as some examples from the cardiac surgery.^[1]^

**Figure 2:**
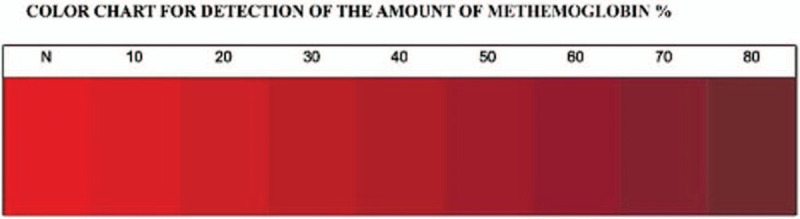
The blood color spectrum of the level of methemoglobinemia% (our case had 80%). (Shihana et al).^[3]^

Met-Hb can be characterized by its color discoloration as brownish^[2]^ and the severity may be shown as a spectrum from red to brown^[3]^ (Fig. 2). Its prevalence rates have geographic variation and the associated etiologies.^[4,5]^ When the level of Met-Hb is high, the physiological damage is not only related to toxicology but also to the level of hypoxemia catastrophe at the tissue and cellular level,^[4–7]^ especially when it is complicated by OHCA, CPR, shock, and hypoxemia-reperfusion injury.

Even a timely diagnosis is made, the management of acquired severe Met-Hb is still challenging, regardless methylene blue is available or not.^[4–7]^ It would usually be fatal due to persistent hypoxemic injury and multiple organ failure despite of maximal ventilator support especially when the antidote is not delivered timely.^[4–7]^ In addition to the conventional treatments reported in the previous literature, VV-ECMO can be used as a bridging therapy before antidote arrives to minimize hypoxemic injuries. The beneficial effect comes from the hyper-oxygenated blood provided by the ECMO much higher than the sole use of ventilator. The hyper-dissolved oxygen, reflected by the very high arterial PO_2_, whereas not the oxygen transported by hemoglobin (impaired by Met-Hb), is the only source available for tissue and cells. Thus, the hyper-oxygenation provided by the VV-ECMO becomes a key factor to the timely and successful management of this case.

This is the first case report for the application of VV-ECMO on salvaging severe acquired Met-Hb. Acquired Met-Hb is uncommon in Taiwan and adjacent countries. It must be diagnosed timely from its unusual presentation and should be treated properly with antidotes and hyper-oxygenation support. If hemodynamics is stable, VV-ECMO should be placed to provide hyper-oxygenation that counter-acts the detrimental effect of Met-Hb.

## Acknowledgment

The authors thank all the staff members that involved in our ECMO program including (not limited to) the Emergency Medical Technicians, Emergency Department, Toxicity Laboratory, and Intensive Care Unit. Antidote was supported by Taichung Veterans General Hospital and they appreciated their help with their most sincere gratitude.

## Author contributions

**Conceptualization:** Yu-Hsuan Lien, Robert Jeenchen Chen.

**Funding acquisition:** You-Cian Lin.

**Supervision:** You-Cian Lin, Robert Jeenchen Chen.

**Writing – original draft:** Yu-Hsuan Lien.

**Writing – review & editing:** Robert Jeenchen Chen.
